# Weaning

**Published:** 1908-05

**Authors:** H. McHatton

**Affiliations:** Macon, Ga.


### Journal-Record of Medicine

Successor to Atlanta Medical and Surgical Journal, Established 1855, and Southern Medical Record, Established 1870.

**OWNED BY THE ATLANTA MEDICAL JOURNAL CO.**

**Published Monthly.**

*Official Organ Fulton County Medical Society, State Examining Board, Presbyterian Hospital, Etc.*

EDGAR G. BALLENGER, M. D., Editor.
BERNARD WOLFF, M. D., Supervising Editor.
A. W. STIRLING, M. D., C. M., D. P. H., J. S. HURT, B. Ph., M. D., Associate Editors.
GEORGE BROWN, M. D., Business Manager.

COLLABORATORS

DR. W. F. WESTMORLAND, General Surgery.
F. W. McRAE, M. D., Abdominal Surgery.
H. F. HARRIS, M. D., Pathology and Bacteriology.
E. B. BLOCK, M. D., Diseases of the Nervous System.
MICHEL HOKE, M. D., Orthopedic Surgery.
CYRUS W. STRICKLER, M. D., Legal Medicine and Medical Legislation.
E. C. DAVIS, A. B., M. D., Obstetrics.
E. G. JONES, A. B., M. D., Gynecology.
R. T. DORSEY, Jr., B. S. M. D., Medicine.
L. M. GAINES, A. B., M. D., Internal Medicine.
J. N. LeCONTE, M. D., Diseases of the Stomach and Intestiness.
L. B. CLARKE, M. D., Pediatrics.
EDGAR PAULIN, M. D., Opsonic Medicine.
R. R. DALY, M. D., Medical Society.
A. W. STIRLING, M. D., Etc., Diseases of the Eye, Ear, Nose and Throat.
BERNARD WOLFF, M. D., Diseases of the Skin.
E. G. BALLENGER, M. D., Diseases of the Genito Urinary Organs.

VOL. X. MAY, 1908. No. 2

### WEANING.*

BY H. MCHATTON, M. D., MACON, GA.

Mr. President and Gentlemen:—

You will probably agree with me that this subject is of extreme importance in the daily work of the average practitioner, and at the same time, one that is most sadly neglected.

Its importance is greater to the little patient than anything that has ever occurred in his life.

In my opinion, more children die from the effects of improper weaning than from all contagious diseases combined. I might with safety say from all causes during the same period of time.

Taking into consideration the vast importance of this neg-

*Read before the Georgia Medical Association, Fitzgerald, April 15, 16, 17, 1908.

lected subject, how many of us are prepared, off hand, to give full and specific instructions to the mother of a child of six, nine or twelve months in regard to its weaning.

There are things that we know are facts, and about which there is no question, in regard to children's food.

1st. That up to a given time there is no food comparable to the milk from the mother's breast.

2nd. This failing, the next safest is a wet nurse.

3rd. If we are willing to accept the concensus of opinion of the specialist in pediatics all over the world, the next safest food obtainable are the various modifications of fresh cow's milk.

4th. That the milk from the Jenny or milk direct from the udder of the goat, as I have seen it much used in the tropics, is good, but practically unobtainable in our work.

5th. That there is no prepared food in the market which can be given alone and continuously for any length of time with safety.

This was proven most conclusively some years ago when a wave of enthusiasm swept over this country in regard to the use of prepared foods, which was followed by a wave of infantile scurvy, such as the oldest practitioner at that time had never seen, and it is to be hoped will never see again.

All of us interested in children's diseases saw more or less cases, almost exclusively in our best families, where our instructions in regard to infant's feeding were most thoroughly carried out.

To this class of foods, I wish to add, only to condemn, ordinary, canned condensed milk. This food usually gives you a beautiful, fat, pink and white baby, ideal to look at from a non-professional standpoint, but very prone to rickets and with absolutely no resisting power when any illness supervenes.

I recently saw some German statistics, which stated that of ten children taken from the mother's breast at birth, one would be alive at the end of the year; that of ten who were nursed exclusively during the first year, nine would be alive.

In fact, each month that the infant can be nourished, even partially, by the mother, adds materially to its chances of surviving.

In regard to the time of weaning or beginning supplementary feeding, each case is a law unto itself. There is no time when feeding should begin, but there are emphatically times when it should not. No child should begin to take artificial food in the hot season when gastro intestinal troubles are so prevalent and so fatal. This rule should be ignored only in the face of the most dire necessity. If it is possible to carry a child through this season on short rations of natural food, and no gain in weight, we should be satisfied.

No child should be suddenly weaned when there is any other recourse. This procedure is always dangerous, and in the summer season, often fatal.

My rules in regard to weaning and supplementary feeding are as follows:

When a baby is born, I give the mother a weekly weight chart, tell her to weigh the baby each week and never give it anything but breast milk and water without instructions.

This request will be carried out about one time in twenty, no matter how much you insist on its importance, but every little helps; and after the baby is made sick a few times, you will get more co-operation from the mother.

As long as the infant is making a normal weekly increase, it is getting enough nourishment. If it is less than one year old, and the mother is well, no insistance on the part of the family should induce you to feed it.

On the other hand, when two or three weeks pass without a gain, or with even a loss, nutrition is not what it should be, and we must find out the cause.

If the infant's digestion is good, we must investigate the condition of the mother, and if nothing can be done to increase the quantity or quality of her milk, the time has arrived for feeding.

This is begun by giving the infant one bottle a day of a modification of milk appropriate to its age.

After taking this for a week, if there are no signs of indigestion, we can give two bottles, following the same rule, and gradually increasing to three or as many as may be required in the given case to produce the normal gain in weight, it being understood that each bottle takes the place of a nursing period.

By following this simple line, we seldom have any trouble. A slight indigestion can usually be controlled by decreasing the quantity or strength of the artificial food, and in unusual cases, a return to the breast milk exclusively until all signs of indigestion have disappeared. Then begin the entire process over again. By this method, we have the inestimable aid of the breast milk until the infant has become used to the artificial food. This plan should be followed with infants of any age up to one year, which is about the time when the average child should be weaned. Children who have been exclusively breast fed can not take as strong solutions of modified milk as those of the same age who have been bottle fed.

I usually begin with a modified milk formula representing one-half or one-third of age of the child. It is a great deal safer to begin too low than too high, and much more scientific to avoid an indigestion than to cure it.

It is perfectly reasonable that a stomach that has dealt with nothing but the most perfect and God-given food should temporarily rebel at any artificial food that mere man, no matter how scientific, can produce.

Last November I had in one office hour two infants, one twelve and one thirteen months old, both magnificent specimens, who had never had any medicine or any food but breast milk, ideal age, children and season.

I gave the two mothers explicit orders as to the preparation of the modified milk. They, as unfortunately is so often the case, ignored my directions completely and put the children on plain milk. Each had an explosion in less than a week; and notwithstanding the fact that in each case, I had an abundance of excellent breast milk, it took me at least a month to get the infants in proper shape again.

These two cases only go to prove that no matter what is the age or condition of the child, or season of the year, we must always be careful when we begin artificial food with an infant.

Mothers will often come to you and announce the fact that their milk did not agree with the infant, and that several weeks ago they weaned it, since which time, they have been unable to get any food that would agree with it.

As this catastrophe usually occurs about the time they have begun to feed the child on any old thing, from sweet potatoes to grated ham, it is much more rational to attribute the existing conditions to the new food than to a sudden alteration in the original food that had agreed perfectly with the infant all of its previous life.

This line of reasoning seems so simple that you are more than surprised when you find that you cannot convince the mother that it is true.

The first thing to do in these cases is to try to re-establish the flow of milk. If this is successful, we are lucky; if not, we are in for a serious and often a fatal condition.

Another cause of a large mortality is the prevalent superstition that the milk of the pregnant woman is, as they express it, "poison to the child."

This superstition is so general that it causes the death of an untold number of children each year. They, the mothers, never wait to seek advice when they think they are pregnant, which proves in most instances not to be the case, but immediately and totally wean the child. It is usually some days before the explosion occurs, then more time is lost before advice is sought, on account of the sister superstition that a teething child should have loose bowels. The mother's milk is usually lost, and we are in for a serious and often a fatal condition.

How this prevalent superstition so at variance with all the laws of nature and presumably of Gad ever originated, or became so prevalent in a Christian country, is an enigma to me; but it is so thououghly disseminated, that few of us who have much to do with sick children go a year without seeing death caused by it.

In the interest of the infant, I never advise weaning simply because pregnancy has occurred; but in the interest of the mother and unborn babe, it is often required,—the majority of our women not being physically capable of standing the double drain of pregnancy and lactation—sudden, complete weaning, never; invariably observe the usual gradual method.

In some countries, it is the common custom to continue nursing during the entire pregnancy, and I have had a case where the mother nursed the child not only during the entire pregnancy, but after the birth of the second child, and no harm ensued to either of the three.

In one rather large and healthy family in my practice, in every pregnancy but the last, the mother was nursing the previous child when quickening occurred.

One of the great troubles in securing a proper compliance with our orders in regard to feeding is that they are often too complicated, and require an amount of education and special training to carry out that is not possessed by those in charge of the infant.

It may be more learned and more scientific for us to give directions about the varying fat percentages of the different layers of the top milk, throw in a few sentences about proteids, fats, carbo hydrates, æsine, etc., but what information does this convey to the anxious mother? She wants to know definitely what to give the infant and how to prepare it. If she cannot get this information from us in a manner that is clear to her, she will seek it elsewhere, and obtain it usually from those banes of our existence, the grandmother and the old, experienced negro nurse, who know more than any doctor about babies, at least in the opinion of the average mother.

To obviate this condition, I have been in the habit of using the following formulas for more than a quarter of a century with complete satisfaction. They may not be perfect, according to the latest laboratory investigations, but they are as near perfect as anything we can get carried out in private work; and what is more, so simple that anyone of ordinary intelligence can comply with the directions. I have used them continuously for over twenty-five years in both hospital and private work, and must say that practically every time I have sought new gods and gone out of this line, with the appropriate modifications to meet special indications in individual cases, it has been to my sorrow.

If any criticism can be made, it is that they are nearer the maximum than the minimum of the amount that the average infant should have.

As given below, they are intended for infants wholly bottle fed, and should be reduced as stated above in this paper.

To all the following tables lime water or soda solution should be added in the proportion of a tablespoonful to a pint. If soda solution is used, add before sterilization; if lime water, after sterilization.

All milk mixtures *must* be sterilized.

Diet during the first week:

| | |
|---|---|
| Cream .................................... | 2 teaspoonfuls. |
| Milk ...................................... | 3 teaspoonfuls. |
| Water (hot) ............................... | 3 teaspoonfuls. |
| Milk Sugar ................................ | ¼ teaspoonful. |

For each portion: to be given every two hours from 5 A. M. to 11 P. M., and in some cases, once or twice at night.

Diet from the second to the sixth week:

| | |
|---|---|
| Milk ...................................... | 1 tablespoonful. |
| Cream .................................... | 2 teaspoonfuls. |
| Milk Sugar ................................ | ¼ teaspoonful. |
| Water ..................................... | 2 tablespoonfuls. |

For one portion: to be given every two hours from 5 A. M. to 11 P. M.

Diet from the sixth week to the end of the second month:

| | |
|---|---|
| Milk ...................................... | 2½ tablespoonfuls. |
| Cream .................................... | 1 tablespoonful. |
| Milk Sugar ................................ | ½ teaspoonful. |
| Water ..................................... | 2½ tablespoonfuls. |

For each portion: to be given every two hours.

Diet from the beginning of the third month to the sixth month:

| | |
|---|---|
| Milk ...................................... | 5 tablespoonfuls. |
| Cream .................................... | 1 tablespoonful. |
| Milk Sugar ................................ | 1 teaspoonful. |
| Water ..................................... | 2 tablespoonfuls. |

For each portion: to be given every 2½ or 3 hours.

Diet during the sixth month: Six meals daily from 6 to 7 A. M. to 9 or 10 P. M.

Morning and mid-day bottles each:

| | |
|---|---|
| Milk ...................................... | 9 tablespoonfuls. |
| Cream .................................... | 1 tablespoonful. |
| Mellins Food .............................. | 1 teaspoonful. |
| Water (hot) ............................... | 2 tablespoonfuls. |

Dissolve the Mellin's Food in the hot water, and add, with stirring, to the previously mixed milk and cream.

Other bottles each:

Milk .................................... 9 tablespoonfuls.
Cream .................................... 1 tablespoonful.
Milk Sugar .................................... 1 teaspoonful.
Water .................................... 2 tablespoonfuls.

In the seventh month, the Mellin's Food may be increased to two teaspoonfuls and given three times daily.

Throughout the eighth and ninth months, five meals a day will be sufficient.

First meal at 7 A. M.:

Milk .................................... 13 tablespoonfuls.
Cream .................................... 1 tablespoonful.
Milk Sugar .................................... 1 teaspoonful.
Water .................................... 2 tablespoonfuls.

Second meal at 10:30 A. M.: milk, cream and water in the same proportions; Mellin's Food, one tablespoonful.

Third meal at 2 P. M. Same as second.

Fourth meal at 6 P. M. Same as second.

Fifth meal at 10 P. M. Same as first.

Mellin's Food may be substituted by Oat Meal or Barley.

### SODA SOLUTION.

Dissolve one drachm of bicarbonate of soda in a quart of boiled water.

This solution will keep indefinitely if well corked, and a tablespoonful of it equals a tablespoonful of lime water in alkalinity.

### RULES FOR STERILIZING.

*First Method.*

Buy an Arnold's Sterilizer and follow attached directions.

*Second Method.*

First. The milk, cream, milk sugar and water for twenty-four hours should be mixed, as soon as received, in a clean, scalded vessel.

Second. The vessel with its contents should be set in a kettle (or sterilizer) of boiling water and allowed to steam twenty minutes.

Third. Pour into the required number of sterilized bottles.

Fourth. Cork the bottles while hot, with cotton, which should not come in contact with the milk. Raw cotton preferred to absorbent.

Fifth. After cooling, place on ice to keep.

*Third Method.*

First. Prepare mixture for the entire day.

Second. Put required amount in each bottle and cork with cotton, which must not come in contact with the milk. Raw cotton preferred to absorbent.

Third. Put bottles in deep tin pail filled with water to neck of bottles. Place on stove and allow to remain for twenty minutes after the water begins to boil.

CARE OF BOTTLES AND NIPPLES.

Cylindrical Nursing Bottles with wide mouths preferred. The bottles should be washed thoroughly in cold water immediately after feeding and set aside full of soda solution.

They should be sterilized immediately before being filled for the next feeding.

Never warm over half used bottles of milk for second feeding—use a fresh bottle.

Plain black rubber nipples, which slip over the neck of the bottle, preferred.

If the hole in the nipple is too small, enlarge by pushing through it a red hot wire. Nipples should be rinsed thoroughly and brushed externally and internally in cold water after feeding, and kept constantly in solution of bi-carbonate of soda.

Very often, in summer, vomiting or acute indigestion is caused by unclean nipples or bottles.

Superstitions are proverbially hard to kill. Among the hardest that I have ever come in contact with is that the grandmother, or an ignorant negro nurse, knows far more about how to feed a child than an educated physician, who has spent a life time in the study of this problem, who has not only his own experience to draw from, but that of the thousands of scientific minds who have preceded him, or who are his co-temporaries along this line of study, and whose combined experiences and deductions were obtained from the close and accurate study of hundreds of millions of babies.

Maybe the time will come when our relative positions will be reversed.

If it ever does, it will surely be a day of hallelujah and rejoicing in babyland.

